# Androgen Receptor Targeted Therapy + Radiotherapy in Metastatic Castration Resistant Prostate Cancer 

**DOI:** 10.3389/fonc.2021.695136

**Published:** 2021-09-23

**Authors:** Maria Massaro, Giuseppe Facondo, Gianluca Vullo, Anna Maria Aschelter, Alessandro Rossi, Vitaliana De Sanctis, Paolo Marchetti, Mattia Falchetto Osti, Maurizio Valeriani

**Affiliations:** ^1^ Department of Radiation Oncology, “Sapienza” University, Sant’Andrea Hospital, Rome, Italy; ^2^ Department of Oncology, “Sapienza” University, Sant’Andrea Hospital, Rome, Italy

**Keywords:** oligo-progressive castration-resistant prostate cancer, androgen receptor targeted therapy, metastasis-directed radiation therapy, conformal radiotherapy, stereotactic body radiotherapy (SBRT)

## Abstract

**Objectives:**

To investigate whether radiotherapy as metastasis-directed therapy (MDT) on oligo-progressive sites in metastatic castration-resistant prostate cancer (mCRPC) patients during treatment with androgen receptor-targeted therapy (ARTT) may lead to control resistant lesions, prolonging ARTT. We analysed progression free survival, overall survival and prognostic parameters that can identify patients that best suit to this approach.

**Patients and Methods:**

Retrospective analysis of a total of 67 lesions in 42 mCRPC patients treated with ablative or palliative RT to oligoprogressive lesions during ARTT. Twenty-eight patients (67%) underwent ARTT with Abiraterone acetate and 14 patients (33%) underwent ARTT with Enzalutamide. Median time between the start of ADT and ARTT beginning was 50.14 months (range 3.37-219 months). We treated 58 lesions (87%) with 3D conformal radiotherapy (3DCRT) and nine lesions (13%) with stereotactic body radiotherapy (SBRT). The Kaplan Meier method was used to assess the median overall survival (OS) and the progression-free survival (PFS).

**Results:**

Median follow-up was 28 months (range 3-82 months). Median OS was 32.5 months (95% CI 25.77-39.16), 1 and 2-year OS were 71.6% and 64.1%, respectively. Median PFS was 19,8 months (95% CI 11.34–28.31), 1 and 2-year PFS were 67.2% and 47.4%, respectively. Median OS for patients that underwent radiotherapy before 6 months from the start of ARTT was 23.4 months (95% CI 2.04-44.89) and 45.5 months (95% CI 31.19-59.8) for patients that underwent radiotherapy after 6 months (*p = 0.009*).

**Conclusion:**

Local ablative radiation therapy directed to progressive metastasis is a non-invasive, well tolerated treatment with efficacy on prolonging clinical benefit of systemic therapies with ARTT. Patients who underwent RT >6 months from the start of ARTT presented a statistically better OS and PFS compared with patients who underwent radiotherapy <6 months from the start of ARTT.

## Introduction

Due to the increased sensitivity and specificity of modern imaging modalities, the oligometastatic prostate cancer (PC) is diagnosed more often, thus many patients considered non-metastatic on conventional imaging (computed-tomography (CT) and bone scintigraphy) turn out to be oligometastatic even at a low prostate-specific antigen (PSA) serum level.

There is currently no definite biologic definition of oligometastatic disease, that is thus defined relying on clinical and radiographic evidence, including the number of lesions and number of sites of metastasis. Oligometastatic PC encloses three different clinical entities (synchronous, metachronous and oligoprogressive cancer) to which castration-resistant (CR) status is added.

According to the European Association of Urology guideline, castration resistance is defined by biochemical progression (three consecutive rises in PSA 1 week apart resulting in two 50% increases over the nadir, with PSA >2 ng/ml) or radiological progression (appearance of two or more new bone lesions on bone scan or enlargement of a soft tissue lesion using RECIST 1.1) with a serum testosterone <50 ng/dl or 1.7 nmol/l (1, 2). This definition is similar to the Prostate Cancer Working Group 3 (PCWG3) criteria published by Scher in 2016 (3).

Despite there is no consensus regarding standard of care treatment for oligometastatic prostate cancer, there is increasing evidence highlighting the importance of local ablative treatments in this setting. A subgroup of patients with mCRPC show oligoprogression, defined by PCWG3 as the first evidence of one new lesion or increased volume of one single existing lesion (3). Always according to PCWG3 about oligoprogressive mCRPC management if multiple sites of disease continue to respond but one to two sites grow, focal therapy such as radiation or surgery could be administered to the resistant site(s) and systemic therapy continued.

Although in most cases PC is initially sensitive to androgen-deprivation therapy (ADT), the majority of PC will develop castration resistance, mostly within 18-24 months from the start of ADT in men with metastatic disease (4). Multiple mechanisms underlying the CR state, include an increased androgen biosynthesis in the tumor microenvironment, androgen receptor (AR) amplifications and overexpression (5).

However, the AR pathway is often found to be activated in mCRPC, meaning that AR itself and its signaling can be still a therapeutic target in this setting of patients (6).

Nowadays the available and approved therapeutic options for the treatment of mCRPC are: abiraterone, enzalutamide, docetaxel, cabazitaxel, radium-223 and sipuleucel-T. Abiraterone acetate (AA) is a selective inhibitor of cytochrome P450 17α-hydroxylase/17,20 lyase (CYP17), interfering with androgen biosynthesis. AA was approved for the treatment of mCRPC after docetaxel in 2011 and for the treatment of mCRPC without previous chemotherapy in 2013 (7–10). The effectiveness of AA was confirmed in 2015 in a randomized phase 3 trial with a median follow-up of more than 4 years showing a benefit in overall survival (OS) and a favourable safety profile (11). Enzalutamide is a second-generation nonsteroidal AR direct inhibitor. It was approved for mCRPC patients after docetaxel in 2012 by U.S Food and Drug Administration (FDA) and 2013 by European Medicines Agency (EMA) and later for chemo-naïve patients in 2014 (12, 13). Also, enzalutamide continues to demonstrate an improved survival in patients with asymptomatic or mildly symptomatic mCRPC, with more than 5 yrs. of follow-up (14).

Even though abiraterone and enzalutamide have radically changed the treatment of mCRPC, a proportion of patients still experience primary and secondary resistance to these novel antiandrogens. Multiple resistance mechanisms have been identified including alterations in AR signaling and AR-independent mutations like neuroendocrine transformation and immune Programmed Death-Ligand 1 (PD-L1) related upregulation. Targeting these pathways will be pivotal for patients with refractory mCRPC (15).

For this reason, novel therapeutic approaches are under clinical investigation, including next-generation AR axis-targeting treatments, immunotherapeutic (PD-1 inhibitors) or targeted-therapies (poly ADP ribose polymerase (PARP) pathways inhibitors) (5).

Clonogens resistant to the current systemic therapy are of paramount importance for the onset of new macroscopic metastasis. The biologic rationale of radiotherapy as metastasis-directed therapy (MDT) is that treating resistant lesions, it is possible to take account to cancer resistant clones *via* multiple mechanisms (as direct killing and immune-mediated cytotoxicity) while continuing Androgen Receptor Targeted Therapy (ARTT) to keep responsive or stable lesions suppressed (16). Additionally, RT may act not only on irradiated lesions but also on other distant metastatic sites, resulting in the abscopal effect. This possibility has already been shown in other solid tumors (17–19).

Several studies have demonstrated improvements in local control (LC), progression-free survival (PFS) and overall survival (OS) with MDT of synchronous, metachronous and oligoprogressive mPC, preserving the current therapy (20–25).

Therefore, the aim of our mono-institutional retrospective experience is to prove that MDT on oligo-progressive sites may allow to achieve local control of lesions resistant to ARTT and thus prolong its use after oligo-progression. We also analysed progression free survival, overall survival and prognostic parameters that can identify patients best suited to this approach.

## Materials and Methods

From 2014 to 2020 we treated 42 oligoprogressive mCRPC patients during androgen receptor targeted therapy (ARTT). The oligoprogressive disease was defined as the radiological evidence of 1–3 new metastasis. Radiotherapy was performed with ablative or palliative intent. We treated a total of 67 lesions in 42 patients, with a median age at ARTT onset of 76 years (range 53–93 years) and median time between the start of ADT and ARTT beginning of 50.14 months (range 3.37-219 months). Only 3 patients (7.1%) developed castration resistance <12 months from the start of ADT.

Twenty-eight patients (67%) underwent ARTT with AA and 14 patients (33%) underwent ARTT with Enzalutamide. Twenty-seven patients (64%) received ARTT as first line treatment, 12 patients (29%) received previously chemotherapy (docetaxel or cabazitaxel) and ARTT as second line therapy. Two patients (4.7%) and one patient (2.3%) underwent ARTT as third- and fourth-line treatment, respectively.

All patients showed a rising PSA level during ARTT and underwent a diagnostic exam that revealed an oligo-progressive disease (from one to a maximum of 3 lesions). The treated lesions were detected by F-18 Fluor Choline-Positron emission tomography–computed tomography (PET-TC) scan or Gallium-68 prostate-specific membrane antigen (PSMA) PET-CT scan and represented the only sites of oligoprogressive metastatic disease for all patients. When indicated, a Magnetic Resonance Imaging (MRI) was performed.

Radiotherapy was performed in 29 patients (69%) on a single disease site, in 7 patients (17%) on 2 lesions and 6 patients (14%) on 3 lesions. Among the 67 metastasis, 58 (87%) were bone lesions, 7 lesions (10%) were located in lymph nodes (lumbar-aortic, external and internal iliac nodes) and 2 (3%) were located in lungs. We treated 58 lesions (87%) with 3D conformal radiotherapy (3DCRT) and nine lesions (13%) with stereotactic body radiotherapy (SBRT). We chose SBRT technique for lung metastasis and focal bone metastasis without involvement of posterior wall of vertebral body.

All methods were carried out in accordance with relevant guidelines and regulations and the study was approved by the internal review board. Each patient analysed in this study gave written informed consent prior to the treatment. Patients’ characteristics are summarized in **Table 1**.

**Table 1:** Patients characteristics (n = 42).

	Details	Patients
Age	Median (range), years	76 years (range 53-93)
Time between ADT and ARTT	Median (range), months	50.14 months (range 3.37-219)
ARTT	Abiraterone	28 (67%)
	Enzalutamide	14 (33%)
Treatment line	I	27 (64%)
	II-III-IV	15 (36%)
RT techniques	3DCRT	58 lesions (87%)
	SBRT	9 lesions (13%)
ARTT and RT	< 6 months	26 (62%)
	> 6 months	16 (38%)
Number of lesions	1	29 (69%)
	2	7 (17%)
	3	6 (14%)
RT sites	Bones	58 lesions (87%)
	Lung	2 lesions (3%)
	Lymphnodes (lumbar-aortic, esternal and internal iliac stations)	7 lesions (10%)

ADT, Androgen deprivation therapy; ARTT, Androgen receptor targeted therapy; RT, Radiotherapy; 3DCRT, Three-dimensional conformal radiation therapy; SBRT, Stereotactic body radiation therapy.

All patients underwent a simulation CT (2.5 mm slice thickness) in the supine position.

In patients treated with SBRT the gross target volume (GTV) included the metastasis mass as identified on planning CT images and then set-up margins were added with an isotropic expansion of 5 mm to obtain planning target volume (PTV). Planning CT images were fused with F-Choline TC-PET and/or Gallium-68 PSMA PET-CT and/or Magnetic Resonance Imaging to GTV delineation. For patients treated with palliative intent the GTV encompassed the bone or lymph-nodal lesions. Treatment was delivered by a linear accelerator using 6–15 MV photons. The dose prescription to the metastatic disease was different according to the site and the size of the lesion involved, as indicated in **Table 2**.

**Table 2:** RT characteristics.

RT techniques	3DCRT	58 lesions (87%)
	SBRT	9 lesions (13%)
Total dose/fractions related to irradiated site and RT techniques	Bone	
	→ SBRT	27Gy/3fr; 18Gy/3fr; 25Gy/5fr
	→ 3DCRT	20Gy/5fr; 30Gy/10fr
	Lung	
	→ SBRT	54Gy/3fr
	Lymph nodes (lumbar-aortic, external and internal iliac stations)	
	→ 3DCRT	40-54Gy/20fr

3DCRT, Three-dimensional conformal radiation therapy; SBRT, Stereotactic body radiation therapy.

For bone metastases the dose commonly used was 20-30 Gy/5-10 fractions using 3DCRT and 27Gy/3fr, 18Gy/3fr and 25Gy/5fr with SBRT technique, while nodal metastases were treated with 40-54Gy/20fr. Lung metastasis were treated with SBRT in 3 fractions of 18 Gy each for a total dose delivered of 54 Gy.

The follow-up consisted of a PSA level measure the first month after RT while maintaining ARTT then every 3 months. In case of PSA increasing and/or appearance of new symptoms, patients were submitted to a new F-Choline PET-TC scan or Gallium-68 PSMA PET-CT scan. If the new imaging revealed the appearance of new metastatic lesions, a shift to other systemic therapy was performed. Acute and late toxicity events were investigated and scored according to the Common Terminology Criteria for Adverse Events v5.0 (CTCAE).

### Statistical Analysis

The statistical analysis was performed using SPSS vv25. The Kaplan Meier method was used to assess the median overall survival (OS) defined as the time elapsed between the beginning of ARTT and death for any cause or the last follow-up, the progression-free survival (PFS) defined as the time elapsed between the start and the suspension of ARTT for radiological progression or death for any cause, and progression-free survival 2 (PFS2) defined as the time elapsed between the radiation treatment and the suspension of ARTT for radiological progression or death for any causes. Sub-group analysis was performed stratifying patients treated with RT < 6-month *vs* > 6-months from the start of ARTT, patients who started ARTT within 24 months *vs* over 24 months from the beginning of ADT, patients treated with SBRT *vs.* 3DRT, patients who received ARTT as first line *vs.* as second-third-fourth line and patients treated on a single metastatic lesion *vs* 2-3 lesions. A p-value lower than 0.05 was considered statistically significant.

## Results

Median follow-up was 28 months (range 3-82 months), median overall duration of ARTT treatment was 15.4 months (range 3–69.7 months), median interval time between the start of ARTT and radiotherapy was 5.0 months (range 2-44.4 months) and median duration of ARTT after radiotherapy was 4.7 months (range 2-59.4 months). Thirty-five patients (83.3%) progressed on multiple sites and thus interrupted ARTT and started new systemic therapies; seven patients (16.6%) maintained ARTT until the last follow-up.

**Figure 1:**
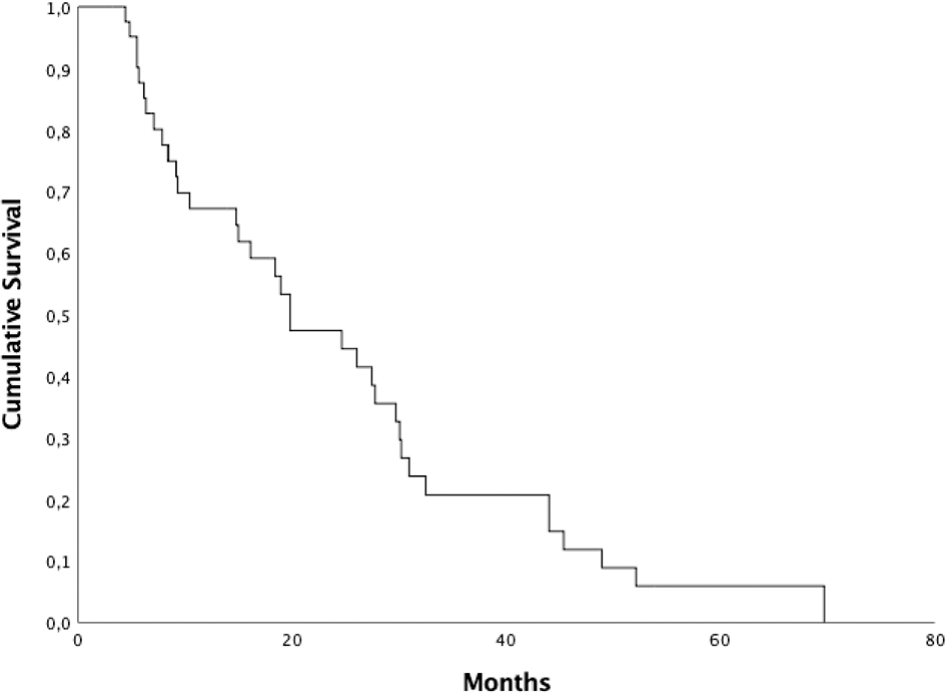
Kaplan-Meyer progression-free survival curve.

Median PFS was 19,8 months (95% CI 11.34–28.31), 1 and 2-year PFS were 67.2% and 47.4%, respectively (**Figure 1**). Median PFS2 was 5,3 months (95% CI 4.25–6.34), 1 and 2-year PFS2 were 38.7% and 19.0%, respectively. Patients who started ARTT within 24 months from the start of ADT presented a median PFS of 27.4 months (95% CI 4.85-49.95) and median PFS2 of 4.8 months (95% CI 3.15-6.44) compared to patients who started ARTT over 24 months from the start of ADT presenting a median PFS of 19.8 months (95% CI 17.75-21.90) (*p = 0.90*) and a median PFS2 of 5.6 months (95% CI 3.27-8) (p = 0.71). Patients who received ARTT with abiraterone acetate presented a median PFS and PFS2 of 26.0 months (95% CI 11.07-40.92) and 5.3 months (95% CI 4.7-5.8), respectively. Patients who received ARTT with enzalutamide presented a median PFS of 19.8 months (95% CI 11.34-31.44) (*p = 0.87)* and a median PFS2 of 13.8 months (95% CI 2.1-29.65) (p = 0.62). Patients who received radiotherapy < 6 and > 6 months after the start of ARTT presented a median PFS of 9.2 months (95% CI 2.12-18.71) and 30.0 months (95% CI 26.98-33.07), respectively (*p = 0.006*). Patients who received radiotherapy < 6 and > 6 months after the start of ARTT presented a median PFS2 of 5 months (95% CI 4.12-6) and 9.5 months (95% CI 1.42-17.63), respectively (p = 0.40). Median PFS and PF2 for patients treated with ARTT as first line therapy was 19.8 months (95% CI 8.37-31.28) and 5 months (95% CI 4.22-5.91) compared to patients treated as second-third-fourth approach 18.4 months (95% CI 7.05–29.80) (*p = 0.79*) and 9.5 months (95% CI 2.44–16.61) (p = 0.62). Median PFS for patients treated with SBRT was 27.7 months (95% CI 2.33-55.12) and 19.8 months (95% CI 12.06-27.59) for those treated with 3DCRT (*p = 0.49*). Median PFS2 for patients treated with SBRT was 4.2 months (95% CI 2.26-6.13) and 5.3 months (95% CI 4.43-6.22) for those treated with 3DCRT (p = 0.82). Patients who underwent RT on a single lesion presented a median PFS and PFS2 of 19.8 months (95% CI 13.76-25.89) and 5.3 months (95% CI 4.41-6.18), respectively. Patients who underwent RT on 2-3 lesions presented a median PFS of 29.6 months (95% CI 13.62-45.71) (*p = 0.30*) and median PFS2 of 5.67 months (95% CI 3.7-7.63) (p = 0.59).

**Figure 2:**
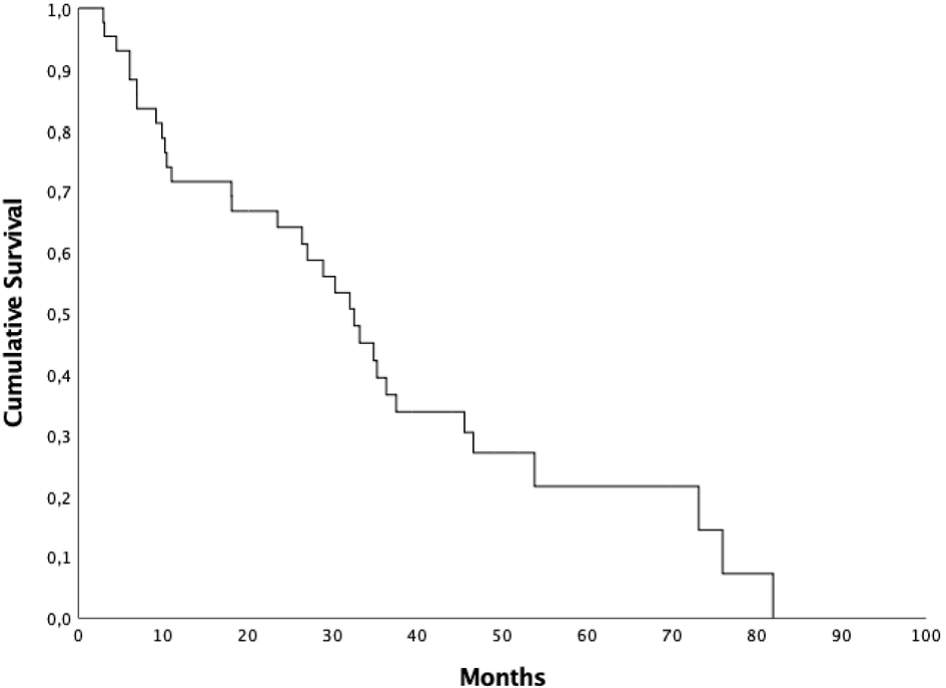
Kaplan-Meyer overall survival curve.

Median OS was 32.5 months (95% CI 25.77-39.16), 1 and 2-year OS were 71.6% and 64.1%, respectively (**Figure 2**). Patients who started ARTT within 24 months from the onset of ADT presented a median OS of 30.2 months (95% CI 4.9-55.56) and patients who started ARTT over 24 months from the onset of ADT presented a median OS of 34.8 months (95% CI 25.4-44.19) (*p = 0.44*). Patients who received ARTT with AA presented a median OS of 32.4 months (95% CI 27.01-37.92) and patients who received ARTT with enzalutamide presented a median OS of 18 months (95% CI 2.1-48.09) (*p = 0.88)*. Median OS for patients treated with ARTT as first line was 32 months (95% CI 26.5-37.43) and for patients treated as second-third-fourth approach was 33.1 months (95% CI 19.63-46.63) (*p = 0.79*). We treated 58 lesions (87%) with 3DCRT and nine lesions (13%) with SBRT. Median OS for patients treated with SBRT was 36.3 months (95% CI 27-45.59) and 30.2 months (95% CI 21.53-38.93) for those treated with 3DCRT (*p = 0.93*).

**Figure 3:**
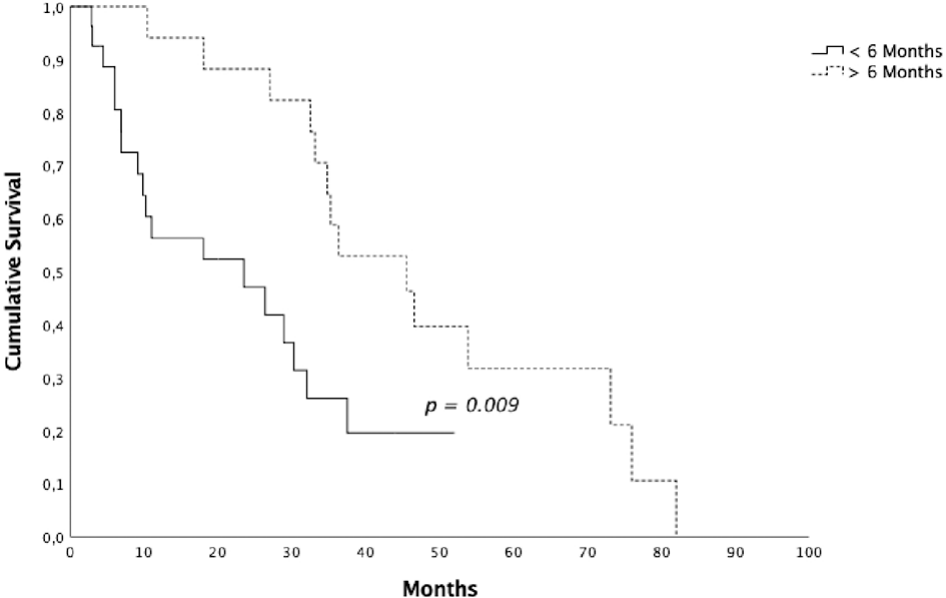
Overall survival for patients submitted to radiotherapy <6 *vs* > 6 months after the start of androgen receptor targeted therapy.

Patients who underwent RT on a single lesion presented a median OS of 29 months (95% CI 17.58-40.21) and patients who underwent RT on 2-3 lesions presented a median OS of 36.3 months (95% CI 0-81.05) (*p = 0.061*). 26 patients (61.9%) underwent radiotherapy before 6 months from the start of ARTT and 16 (38.1%) after 6 months. Median OS for the first group was 23.4 months (95% CI 2.04-44.89) and 45.5 months (95% CI 31.19-59.8) for the second group (*p = 0.009*) (**Figure 3**). Data are summarized in **Table 3**.

**Table 3:** Univariate analysis.

	Median PFS (months)	2-year PFS (%)	p-Value
ARTT line			*0.79*
I line	19.8	48.4	
II-III-IV line	18.4	46.5	
RT technique			*0.49*
SBRT	27.7	60.0	
3DCRT	19.8	45.4	
ARTT and RT			***0.006***
<6 months	9.2	32.1	
>6 months	30.0	67.6	
Number of lesions			*0.30*
1 lesion	19.8	42.1	
2-3 lesions	29.6	61.4	
	Median OS (months)	2-year OS (%)	p-Value
ARTT line			*0.79*
I line	32.0	65.0	
II-III-IV line	33.1	62.7	
RT technique			*0.93*
SBRT	36.3	100	
3DCRT	30.2	59.0	
ARTT and RT			***0.009***
<6 months	23.4	47.1	
>6 months	45.5	88.2	
Number of lesions			*0.061*
1 Lesion	29.0	55.0	
2-3 lesions	36.3	84.6	

ARTT, Androgen receptor targeted therapy; RT, Radiotherapy; 3DCRT, Three-dimensional conformal radiation therapy; SBRT, Stereotactic body radiation therapy. Bold values is the statistically significant value (p < 0.05).

During and after the treatment no toxicities were recorded according to the CTCAE v5.0.

Before radiotherapy 35 patients (83.3%) reported pain (Numerical Rating Scale median value 6, range 1–9); after radiotherapy 21 patients (50%) reported pain with NRS median value of 2 (range 1-7).

## Discussion

In progressive castration resistant prostate cancer, the historical standard of care was based on the interruption of the current therapy and switch to a new line of treatment. Radiotherapy was used collaterally with palliative intent. Radiation therapy may allow to avoid treatment shift when the current therapy still retains efficacy. The effectiveness of radiotherapy for the treatment of synchronous, metachronous and oligoprogressive mPC has been proven by multiple previous experiences.

The aim of this paper is to demonstrate the benefit obtained by radiotherapy as MDT on prolonging the duration of ARTT and so delaying the start of a new line of treatment. In our series of patients, median OS was 32.5 months, 1 and 2-year OS were 71.6% and 64.1%, respectively, and 7 patients (16.6%) maintained ARTT until the last follow-up. Radiation therapy was well tolerated, without reported toxicities and with an optimal pain control.

The safety of concomitant palliative RT to bone sites and abiraterone was investigated by Saad in 2012 in a *post-hoc* exploratory analysis of COU-AA-301 randomized trial (26). The aim of this analysis is to assess the safety and tolerability of abiraterone acetate in combination with radiation therapy in a subset of patients who had localized progression at a single site. Of 1185 patients enrolled in the trial, 136 patients progressed at a single site, and they received concomitant RT. Of these, 42% of patients in the AA group and 25% in the placebo group remained on treatment more than 12 weeks after RT. The median time from the beginning of AA or placebo to RT was 15.1 weeks and 8.7 weeks and median time from first dose of concomitant RT to end of treatment was 8.7 weeks and 7.9 weeks, for AA and placebo groups, respectively. So palliative radiation therapy to bone can be safely performed with abiraterone to patients with localized progression at a single site.

Also, an Italian experience by Detti and colleagues investigated whether the addition of radiotherapy to AA can influence treatment outcomes (27). They treated an unselected population of 32 patients with mCRPC previously treated with docetaxel or deemed unfit to chemotherapy. Patients underwent RT at the median radiation dose of 30 Gy (range 6-58.8 Gy). Most patients (81.3%) received palliative RT mainly on bone, followed by lymph node (9.4%), prostate (6.2%) and visceral (3.1%). Most patients were treated with 3D conformal radiotherapy and no adverse events leading to treatment suspension or discontinuation were reported during treatment. They obtained a PFS after RT of 9.6 months and a median duration of AA treatment after RT of 4.9 months (range 0.2-25.6 months), in line with our experience. Overall, the use of radiotherapy allowed to continue AA for 7 additional months, with a total period on AA of more than 1 year. This therapeutic strategy resulted in very favourable clinical outcomes, with a PFS of about 13 months and an OS of approximately 18 months. Differences between pre-treated patients and patients unsuitable for docetaxel were negligible. MDT in PC has experienced significant advanced with the increasing use of SBRT.

Regarding the SBRT, Nguyen reported a case of a patient with mPC who progressed with a growing solitary metastatic node while on ARTT with enzalutamide, but he went on to have durable complete remission after SBRT to the progressing site of disease, continuing enzalutamide (28). The patient, after an increasing in size of a para-aortic lymph node with a PSA rising, received SBRT 50 Gy in 10 fractions. So SBRT proves to be effective in long-term control of oligoprogression of CRPC with a concomitant decline of PSA.

In addition to the evolution of RT techniques, with the increasing use of SBRT, we also saw the improvement of imaging methods. 68Ga-PSMA PET-CT has demonstrated a high sensitivity and specificity in the detection of nodal, bone and visceral metastasis. In RT planning, 68Ga-PSMA PET-CT can affect dose prescription, target delineation and use of concomitant therapy (29). This successful approach has been proven by Lohaus and colleagues, who have shown the efficacy of PSMA-PET-guided local ablative RT in 15 oligometastatic CRPC patients (30). A relevant subset of patients with 68GaPSMA-PET–detected oligometastatic low-volume CRPC had a meaningful PSA response to local ablative RT. Yoshida analysed a cohort of 101 patients with CRPC who underwent a whole-body DWI-RM, as tumor activity’s parameter (31). Their objective was to evaluate the treatment outcome of progressive site-directed therapy for CRPC. The regional RT targets were the prostate/pelvic nodes, bone or both. In addition to a high benefit with this treatment with PSA response, they identified the pelvis as a good candidate for progressive site-directed therapy. Recently Mazzola and colleagues have compared the impact of 18F-choline and 68Ga-PSMA PET-CT in patients affected by castration-sensitive oligorecurrent PC treated with MDT (32). They analyzed a total of 118 lesions in 88 patients and 44 (50%) patients underwent 68Ga-PSMA PET-guided SBRT while the remaining underwent choline PET-based SBRT. After a median follow-up of 25 months, OS and LC were both 100%. DFS rates were 63.6% and 34%, respectively, in the 68Ga-PSMA and choline PET group (p = 0.06). So PSMA-PET-guided SBRT has allowed a higher rate of ADT-free patients when compared with the 18F-choline-PET cohort.

Beside the benefit of RT on metastatic burden, it has also been shown a better outcome in mCRPC patients treated with curative RT to the primary tumor during abiraterone treatment (33). They retrospective evaluated 106 mCRPC patients; local RT to the primary tumor and pelvic lymphatics was delivered in 44 patients (41%) and 62 patients (59%) did not have RT to the primary tumor. Median OS was higher in patients treated with local RT to the primary tumor than in those treated without local RT with borderline significance (p= 0.08). Patients treated with primary RT had significantly less progression under AA (p=0.03) and a longer AA period than those treated without local prostate RT (p= 0.04). Improvement in OS may be due to the elimination of a primary tumor with the possibility to produce metastases, and it may represents the rationale for a definitive treatment of the primary tumor. Furthermore, this approach could help to maintain quality of life while eliminating the symptoms related to local tumor progression. Instead regarding RT on metastatic sites in this setting of patients, over the past five years, emerging retrospective studies have been published on MDT in mCRPC patients, demonstrating the possibility to prolong current systemic treatment.

In a bi-institutional retrospective study, Berghen et al. tested the hypothesis that progression-directed therapy might defer the initiation of next-line systemic treatment (34). Thirty patients with mCRPC experienced oligoprogression, defined as a total of three or fewer progressive lesions either at known metastatic sites and/or the appearance of new metastasis and/or local recurrence. All these patients received an ablative therapy on the progressive lesions, while ongoing systemic treatment was maintained. Treatment of metastatic disease consisted of SBRT, metastasectomy or fractionated RT. Median next-line systemic treatment-free survival was 16 months (95% CI 10–22) and PFS of 10 months (95% CI 6–15) with only minor radiotherapy- or surgery-related toxicity. These results suggest that RT directed to progressive metastatic lesions could postpone the switch to the next line of systemic treatment.

The cohort described by Deek et al. which consisted of 68 patients with oligoprogressive CRPC, was treated with SBRT on a total of 112 lesions including bone, node and visceral sites (35). The median BED3 was 130.0 Gy. The cumulative incidences of local failure at 1 and 2 years were 2.1% and 13.8%, respectively. Compared with change in systemic therapy alone, MDT was associated with improved median time to next intervention (p = 0.025) and distant metastasis-free survival (p = 0.045). These results suggest that radiotherapy of oligoprogressive lesions can result in sustained periods of disease free survival and might add benefit in addition to ARTT at the time of progression.

Recently an Italian retrospective multi-institutional analysis of 34 mCRPC patients treated with SBRT to oligoprogressive lesions during ARTT was published (36). SBRT was delivered to a median total dose of 30 Gy (27–36 Gy) in 3–5 fractions with a BED3>100 Gy in all cases. Median next-line systemic treatment-free (NEST) survival, PFS and OS were 16.97, 13.47 and 38.3 months, respectively. The median OS was 38.3 months, with a 2-year OS of 74.9%. Factors associated with worse NEST-free survival and r-PFS were PSA ≤ 7 ng/ml at mCRPC diagnosis (p=0.017; p=0.006) and PSADT ≤ 3 months at mCRPC diagnosis (p=0.026; p=0.037). Regarding toxicity, no patient developed acute and/or late grade ≥ 2 toxicity. This study highlights the potential for systemic and local treatment integration in the management of mCRPC patients.

One of the latest experience about SBRT was made by Onal and colleagues in Turkey (37). They treated 126 oligoprogressive lesions in 54 metastatic castration‐resistant prostate cancer patients during therapy with abiraterone or enzalutamide, before or after systemic chemotherapy. The dose range was 16 or 18 Gy in single fraction in patients with spine metastasis, 10 Gy per 2 fractions in patients with bone metastasis, 30-40 Gy in 5 fractions for lymph node metastases. The median BED of SBRT was 101.3 Gy (range: 90–146.7 Gy). After a median follow‐up of 19.1 months, The median PFS was 12.7 months (95% CI: 7.2–18.2 months) and the timing of ARTT treatment (before or after chemotherapy) and the prostate‐specific antigen (PSA) response after MDT were significant prognostic factors for PFS. So MDT for oligoprogressive lesions is effective and may provide several benefits compared to switching from ARTT treatment to next‐line systemic treatment. Data from prospective cohorts are eagerly awaited to confirm the promising results reported in these retrospective series.

Therefore, a prospective randomized phase II trial (ARTO trial, NCT03449719) is currently ongoing, randomizing mCRPC patients to receive a first line with abiraterone acetate (control arm) or abiraterone acetate plus SBRT on all sites of disease (experimental arm). There are other ongoing phase II trials investigating the role of SBRT in combination with standard of care therapy, such as FORCE (NCT03556904) and DECREASE trial (NCT04319783). The ongoing phase II FORCE trial randomize men with CR OPCa to systemic therapy ± MDT and the DECREASE trial randomize men to darolutamide ± MDT. Another critical issue that still needs to be clarified is the definition of the subsets of patients that might benefit the most from MTD at disease progression. In our series, patients who underwent RT after 6 months from the start of ARTT achieved better OS and PFS compared with patients who underwent radiotherapy before 6 months from the start of ARTT. Type of radiation therapy, time from the beginning of ADT and castration resistance status, lines of treatment and number of metastatic lesions were not prognostic factors.

### Limitations of the Study

Limitations of our study are the retrospective design with a low number of patients. The absence of a case-control group of patients not receiving RT does not allow us to draw definitive conclusions about MDT role in improving survival outcomes. Heterogeneity of radiotherapy regimes involving a different intent (ablative and palliative) is another limitation. Despite the emerging role of advanced imaging (e.g., F-18 Fluor Choline/PSMA PET-CT scans and multiparametric MRI), CT and bone scans still represent the standard of care in clinical practice, and they are used in most clinical trials for ARTTs. Our approach using advanced imaging for detection and follow-up of oligoprogressive treated lesions may have influenced treatments timing and furthermore survival outcomes.

Further studies are necessary to confirm or not the results of this study and to search other prognostic factor in order to select oligoprogressive patients that might have an advantage from a local treatment without interruption of ARTT in respect to those that need an immediate change of therapeutic strategy.

## Conclusion

Local radiation therapy directed on progressive metastasis is a non-invasive, well tolerated treatment and prolongs clinical benefit of systemic therapies with ARTT. Patients who underwent RT >6 months from the start of ARTT presented a statistically better OS and PFS compared with patients who underwent radiotherapy <6 months from the start of ARTT. Prospective randomized studies and studies with more cases are necessary to confirm our results and to evaluate other prognostic factor in order to select patients with a high benefit from this approach.

## Data Availability Statement

The raw data supporting the conclusions of this article will be made available by the authors, without undue reservation.

## Ethics Statement

Ethical review and approval was not required for the study on human participants in accordance with the local legislation and institutional requirements. The patients/participants provided their written informed consent to participate in this study.

## Author Contributions

MM, GF, GV, and MV were major contributors in writing the manuscript, analysed the data and drafted the article. MV made substantial contributions to conception of the study. AA, AR, VS, PM, and MO provided clinical expertise in this project. All authors contributed to the article and approved the submitted version.

## Conflict of Interest

The authors declare that the research was conducted in the absence of any commercial or financial relationships that could be construed as a potential conflict of interest.

## Publisher’s Note

All claims expressed in this article are solely those of the authors and do not necessarily represent those of their affiliated organizations, or those of the publisher, the editors and the reviewers. Any product that may be evaluated in this article, or claim that may be made by its manufacturer, is not guaranteed or endorsed by the publisher.
